# Novel Uses of the SwiftNinja Steerable Microcatheter for Pediatric Cardiovascular Interventions

**DOI:** 10.1007/s00246-024-03516-7

**Published:** 2024-05-18

**Authors:** Christopher Herron, Shabana Shahanavaz

**Affiliations:** 1 Department of Pediatric Cardiology, Arnold Palmer Hospital for Children, Orlando, FL USA; 2 Department of Pediatrics, University of Cincinnati, Cincinnati, OH USA; 3 Division of Pediatric Cardiology, The Heart Institute, Cincinnati Children’s Hospital, 3333 Burnet Ave, Cincinnati, OH 45229 USA

**Keywords:** Pediatric intervention, Congenital heart disease, Steerable microcatheter

## Abstract

In the present era, the intricacy of procedures undertaken by a pediatric interventional cardiologist has increased, primarily attributed to dealing with smaller, younger patients with more complex anatomies. To adapt to these smaller and more complex patients, we must adapt our interventions and our equipment to perform these procedures. This article outlines various innovative applications of the SwiftNinja steerable microcatheter within the pediatric cardiac catheterization laboratory.

## Introduction

Cases within the pediatric cardiac catheterization lab are becoming increasingly nuanced with smaller patients, more complex anatomies and sicker patients. To successfully intervene in this patient population our equipment must be able to get us to areas that are difficult to access. The SwiftNinja steerable microcatheter (Merit Medical, South Jordan, UT, USA) is a 2.4Fr catheter that has an intraluminal diameter allowing up to a 0.018″ wire and has up to 180-degree directionality. While initially created for adult applications such as percutaneous coronary interventions, neurovascular aneurysms, acute ischemic strokes and peripheral interventions, this catheter is extremely versatile and has many applications in pediatric congenital heart disease patients [1–7]. We present a case based review of the many applications for this steerable microcatheter in the pediatric cardiac catheterization lab.

### PDA Recanalization

**Fig. 1:**
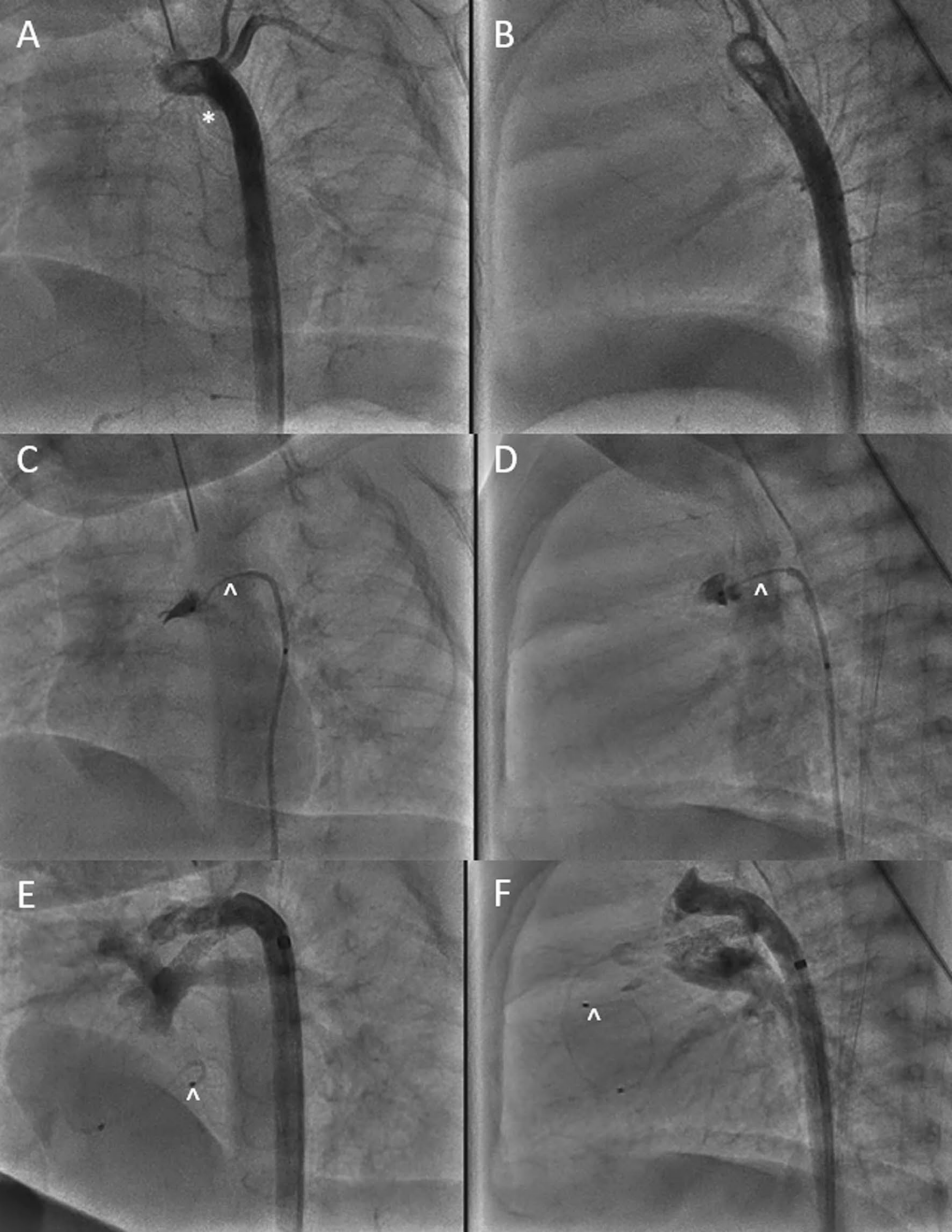
**A**, **B** Initial angiography depicting no PDA present but a small aortic end ampulla is present (*). **C**, **D** The SwiftNinja steerable microcatheter (^) is used no navigate recanalization of the PDA and angiography shows the pulmonary end of the PDA. **E**, **F** The newly recanalized PDA is stented with appropriate flow to the pulmonary arteries. The SwiftNinja steerable microcatheter (^) is positioned within the right ventricle

A 13-day-old male with tricuspid atresia, normally related arteries with severely hypoplastic right ventricle had persistent desaturations with spontaneous closure of his patent ductus arteriosus (PDA). He was brought to the cardiac catheterization lab for attempted recanalization of his PDA. Initial angiography depicted absence of flow through a PDA except for in the small aortic ampulla (Fig. 1A, B). A rail was created using the SwiftNinja steerable microcatheter and a 0.014’ CTO wire through a 4Fr JR catheter. The SwiftNinja allowed us to have steep angulation within the aortic ampulla and direct the wire and traverse the course of the slightly tortuous duct in the direction of the presumed main pulmonary artery (Fig. 1C, D). A wire was then incrementally advanced from the aorta to the main pulmonary artery and into the right ventricle using the SwiftNinja to steer as required. The newly recanalized ductus was then stented using a drug eluding stent (DES) with appropriate retrograde flow into the branch pulmonary arteries (Fig. 1E, F). 5 months later he was able to undergo a successful Bidirectional Glenn palliation.

### Pulmonary Valve Perforation

**Fig. 2:**
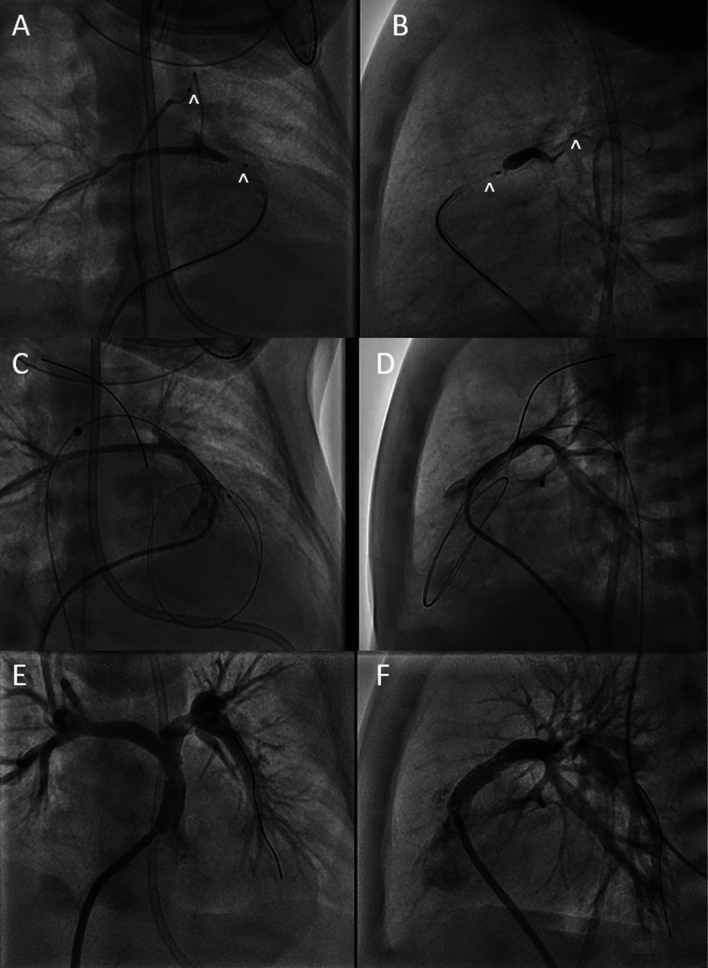
** A**, **B** A retrograde SwiftNinja steerable microcatheter (^) traverses the MAPCA and sits in the main pulmonary artery segment. There is bilateral pulmonary artery hypoplasia. An antegrade SwiftNinja steerable microcatheter (^) is seen in the right ventricular outflow tract as a target for pulmonary valve perforation. **C**, **D** Post retrograde pulmonary valve perforation and balloon dilation showing a communication between the right ventricle and the main pulmonary artery. **E**, **F** Post retrograde stenting of the right ventricular outflow tract with appropriate antegrade flow across the stent into the branch pulmonary arteries

A 2-day-old male weighing 3.5 kg was born with membranous pulmonary atresia, ventricular septal defect and major aorto-pulmonary collateral vessels (MAPCAs). Native pulmonary arteries were present though severely hypoplastic and no PDA was present. Initial angiography revealed 3 large MAPCAs, with 2 of them connecting to the native pulmonary arteries. Initially attempts were made to obtain an appropriate catheter position in the right ventricular outflow tract (RVOT) to perform antegrade perforation but was unsuccessful given the horizontal position and the small size of the target main pulmonary artery. We then proceeded to place the SwiftNinja microcatheter through the left sided MAPCA retrograde into the native main pulmonary artery and one into the right ventricular outflow tract as a perforation target (Fig. 2A, B). A 0.014' CTO wire was advanced through the SwiftNinja microcatheter which helped achieve better alignment given the ability to steer*.* The atretic pulmonary valve was then perforated retrograde and retrograde RVOT stenting was performed using a DES coronary stent. Post intervention angiography showed an unobstructed RVOT with brisk flow into the native pulmonary arteries (Fig. 2C, D). The patient was then brought back to the cardiac catheterization lab for further balloon dilation of the RVOT stent 5 weeks later with interval growth of the native pulmonary arteries (Fig. 2E, F).

### Mee Shunt Recanalization

**Fig. 3:**
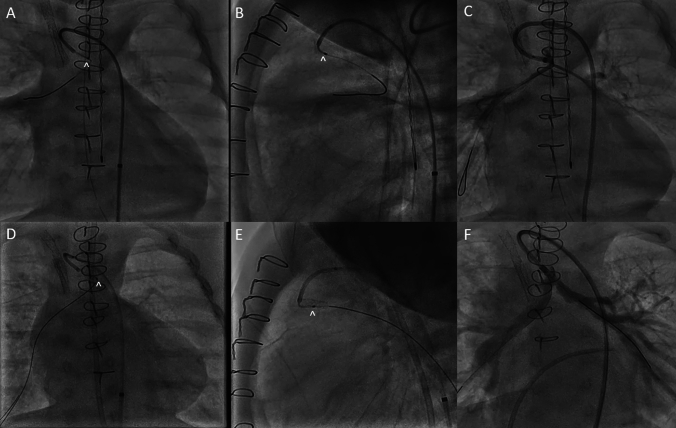
**A**, **B** The SwiftNinja steerable microcatheter (^) assisting in wire position across the Mee shunt in the right pulmonary artery. **C** Post initial balloon dilation of the Mee shunt, angiography depicting severely hypoplastic pulmonary arteries. **D**, **E** The SwiftNinja steerable microcatheter (^) is able to make the 180° turn from the ascending aorta through the Mee shunt and into the right pulmonary artery. **F** Post balloon dilation angiography of the stented Mee shunt and bilateral pulmonary arteries

A 2-month-old male born with complete atrioventricular septal defect, double outlet right ventricle with pulmonary atresia, confluent but severely hypoplastic native pulmonary arteries and MAPCAs underwent a Mee shunt at 1 month of age connecting the native pulmonary arteries to the ascending aorta. No flow was visualized a week post operatively, so the patient was brought to the cardiac catheterization lab for recanalization. Multiple catheter and wire combinations were attempted to gain access to the shunt. Ultimately, the SwiftNinja steerable microcatheter and a 0.014 wire through a 4Fr MPA 2 catheter gained access to the shunt (Fig. 3A, B). The native pulmonary arteries were severely hypoplastic post recanalization (Fig. 3C). The shunt was then stented with a DES coronary stent. The patient was brought back to the cardiac catheterization lab for further dilation of the Mee shunt and native pulmonary arteries. The SwiftNinja steerable microcatheter was again used to gain access to the stented Mee shunt due to the extreme angulation from the ascending aorta (Fig. 3D, E). The shunt and native pulmonary arteries were further balloon dilated with significant improvement to their caliber (Fig. 3F).

### Pulmonary Flow Restrictors

**Fig. 4:**
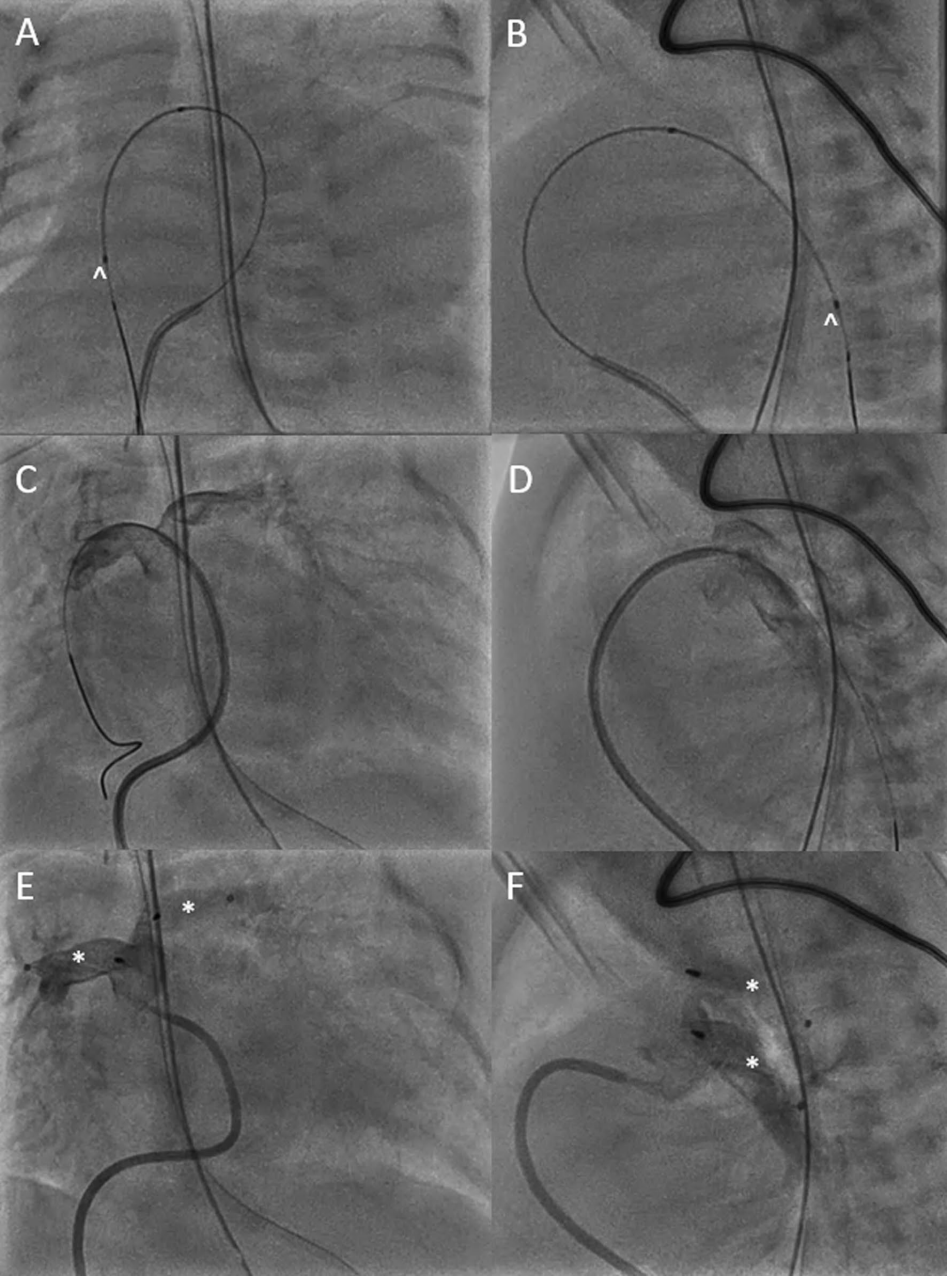
**A**, **B** With the use of the SwiftNinja steerable microcatheter (^) distal wire position was obtained in the right lower pulmonary artery. **C**, **D** Initial pulmonary artery angiography depicting acute angulation of bilateral pulmonary arteries through both appropriate in caliber. **E**, **F** Post flow restrictor placement in the bilateral pulmonary arteries with appropriate flow seen

A 3 week old, 31 week premature female with double outlet right ventricle, subaortic VSD, severe pulmonary stenosis and aortic stenosis along with a large AP window presented for bilateral pulmonary artery flow restrictors due to the patient’s weight of 1.7 kg. Through femoral venous access, a 0.018 wire was able to be placed in the right lower pulmonary artery with the help of the SwiftNinja steerable microcatheter (Fig. 4A, B). Given the acute angulation of the pulmonary arteries and the near pulmonary atresia the SwiftNinja was able to steer the wire into branch pulmonary arteries without the need of stiff guide catheter (Fig. 4C, D). Once the wire and microcatheter were placed distally, a JB1 catheter was able to track into the lower pulmonary artery for device deployment. Medtronic 7Q microvascular plugs (Medtronic, Minneapolis, MN, USA) were placed in both pulmonary arteries successfully (Fig. 4E, F).

### Mechanical Thrombectomy

**Fig. 5:**
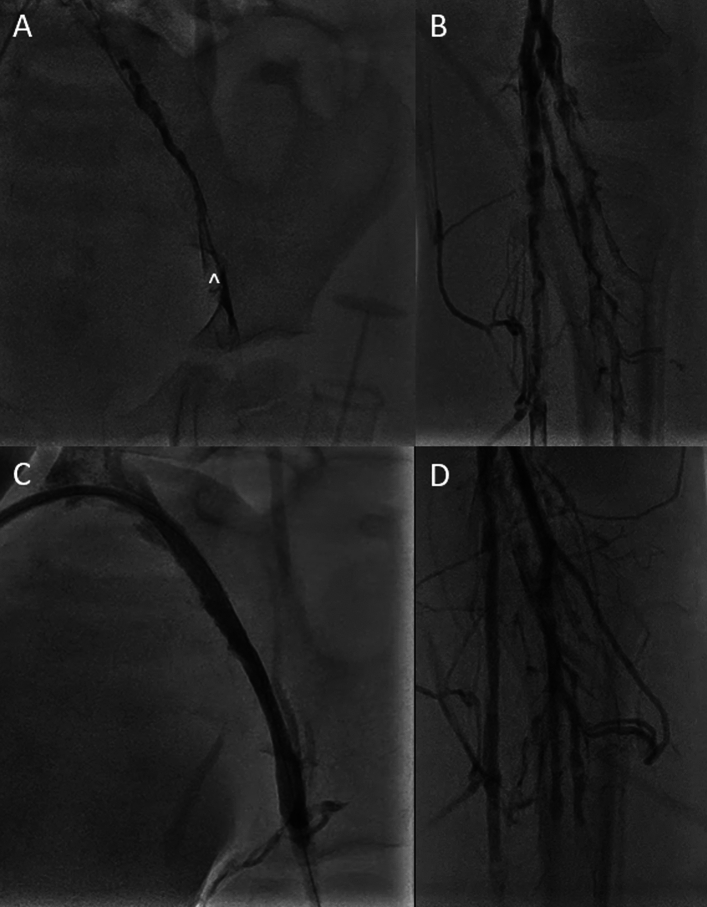
**A**, **B** Angiography within the left common iliac vein down to popliteal vein with extensive thrombus throughout all veins. The SwiftNinja steerable microcatheter (^) is shown within the femoral vein. **C**, **D** Post thrombectomy the left common iliac vein down to the popliteal vein is shown with significantly improved flow and extensively less thrombus burden

A 3-year-old previously healthy female presented with fatigue, lower extremity edema and was found to be extremely anemic with a hemoglobin of 3.7 g/dL due to a diagnosis of very early onset inflammatory bowel disease. A CT scan was performed revealing extensive, near occlusive thrombus from the infrahepatic inferior vena cava down to the bilateral popliteal veins (Fig. 5A, B). She was brought to the catheterization lab for mechanical thrombectomy using the Penumbra Indigo aspiration system (Penumbra Inc, Alameda, CA). Once past the common iliac vein, the SwiftNinja steerable microcatheter was able to navigate the significant thrombus burden and gain distal position. It was particularly useful in the smaller popliteal branches so that multiple branches could be accessed and thrombectomy performed. Post intervention angiography showed significant improvement from the common iliac vein down to the popliteal vein (Fig. 5C, D).

### Premature PDA Device Retrieval

**Fig. 6:**
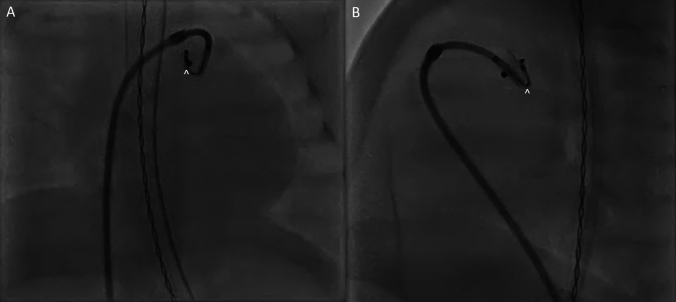
**A**, **B** A embolized PDA device is seen with the main pulmonary artery. Through a 4Fr long sheath, the SwiftNinja steerable microcatheter (^) is able to place a 5 mm microsnare on the pin of the device for capture

A 5-week-old female born prematurely at 27 weeks was found to have a large PDA with left heart dilation. She was brought to the catheterization lab for transcatheter PDA closure. Initially, a 5-2 Amplatzer Piccolo PDA occluder (Abbott, North Chicago, IL) was chosen for the large PDA. The device was deployed, and echocardiogram depicted trivial residual shunt with no left pulmonary artery stenosis or coarctation, so the device was released. The device embolized to the main pulmonary artery just after release. A 4Fr Flexor sheath was introduced into the main pulmonary artery through which a JB1, SwiftNinja steerable microcatheter and microsnare were introduced. Using the directionality of the SwiftNinja steerable microcatheter allowed us to place the snare on the Piccolo pin and retrieve the entire system back into the long sheath successfully (Fig. 6A, B). The ability to grab the pin specifically allows for an easier retrieval when removing the device through a long sheath versus grabbing the body of the device.

## Discussion

The SwiftNinja steerable microcatheter has become our go to microcatheter due to its 180-degree directionality, ability to take a 0.018 wire and the overall ease of use. We have utilized this catheter for a multitude of congenital heart disease anatomies and in a variety of procedures.

Initial reports describe its use in adult patients for percutaneous coronary interventions, ischemic strokes, neurovascular aneurysms and peripheral interventions [1–4]. Hoffmann et al. described their use of the SwiftNinja microcatheter in peripheral vessel interventions and the significant reduction in time to target vessel cannulation and a reduction in radiation exposure [7]. Other unique applications have been described such as embolization of lymphatic vessel chylous ascites, anorectal variceal due to bleeding and a pulmonary artery pseudoaneurysm [8–10].

Pediatric congenital catheterizations are often challenging due to small spaces with tighter turns and the challenges are only augmented by having to use equipment “borrowed” from adult interventionalist which are not made to traverse the pediatric vasculature. Procedural complications in published reports are highest in the neonatal population with younger age and weight associated with higher adverse event rates [11, 12]. While cannulation of vessels in the adult population can be augmented with guide catheters and deflectable sheaths for support and directionality, this avenue is generally not possible in our neonatal population. The stiffness of the catheter can also prop open the tricuspid valve when used in the right heart, which in a neonate can cause significant hemodynamic compromise or rarely can cause vessel perforation with advancement. These reasons have led to the increased use of steerable microcatheters for directionality within vessels. The use of microcatheters likely reduces the duration of the procedure although we did not systematically evaluate the duration of the procedure. This is a very important aspect as duration of procedure was also found to be an independent predictor of outcomes. Catheter based RVOT perforations in neonates with pulmonary atresia and tetralogy of Fallot is considered one of the highest risk procedures especially due to the offset of the RVOT and the main pulmonary artery as well as the inability of catheters to angulate within the anatomy [13–16]. The SwiftNinja steerable microcatheter can be used to guide the CTO wire or RF wire towards the MPA or in retrograde perforations towards the RVOT with more precision.

The recapture of an embolized device is often complicated by the inability to grab the “pin” of the device. Although the device can be snared along its body this can make it challenging to recapture within the sheath and might warrant upsizing the sheath [17, 18]. The pin often might be turned away from the path of approach making it more challenging to grab. The ability of the SwiftNinja steerable microcatheter to move beyond the device due to its small caliber and to achieve a tight curvature to grab the pin can certainly reduce the need for upsizing the sheath or using undue force in device retrieval.

The use of the SwiftNinja steerable microcatheter has been described in the pediatric population for splenic and renal artery embolization and in a pediatric patient with Fontan physiology requiring embolization of a portosystemic shunt [5, 6]. Its use in the pediatric population, however, is not limited to just embolization. As we have described above, its ability to cannulate very small tortuous vessels allows for a multitude of procedures to be performed and can even help rescue the interventionalist from device embolization. The SwiftNinja steerable microcatheter has become our go to microcatheter for a multitude of procedures performed in complex congenital heart disease and in small pediatric patients.

## Conclusion

As the patients a pediatric interventional cardiologist is intervening upon become more complex and smaller, we must adapt our interventions and equipment to suit this population. We describe the use of the SwiftNinja steerable microcatheter in a variety of congenital heart disease anatomies and in a variety of interventional procedures.

## Data Availability

No datasets were generated or analysed during the current study
